# Carbon nitride used as a reactive template to prepare mesoporous molybdenum sulfide and nitride†

**DOI:** 10.1039/d1ra03657b

**Published:** 2021-06-18

**Authors:** Daria Ryaboshapka, Pavel Afanasiev

**Affiliations:** Univ. Lyon, Univ. Claude Bernard Lyon 1, CNRS, UMR5256, IRCELYON F-69626 Villeurbanne France pavel.afanasiev@ircelyon.univ-lyon1.fr

## Abstract

Carbon nitride C_3_N_4_ has been used as a sacrificial template to prepare inorganic materials with hierarchical pore structure. C_3_N_4_ impregnated with ammonium heptamolybdate was treated in reactive gas mixtures (H_2_S/H_2_ or NH_3_/H_2_). This approach allowed mesoporous molybdenum sulfide and molybdenum nitride materials to be obtained that replicate the morphology of the C_3_N_4_ template. Advantageous catalytic properties have been demonstrated in the thiophene hydrodesulfurization (HDS) and electrochemical hydrogen evolution reaction (HER). The highest rates in both reactions were observed for partially sulfidized Mo_2_N solid.

## Abstract 2

Carbon nitride C_3_N_4_ has been used as a sacrificial template to prepare inorganic materials with a hierarchical pore structure.

## Graphical abstract

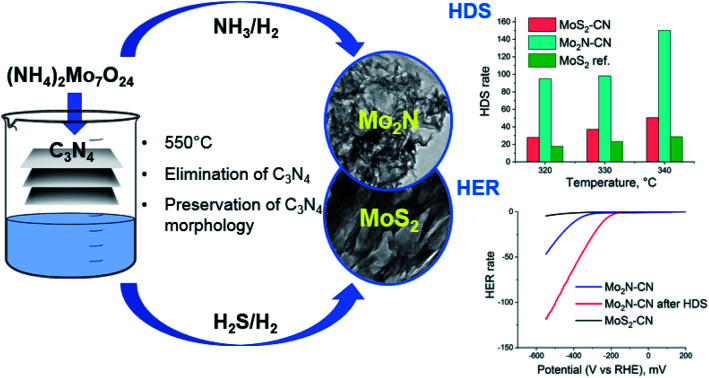

## Introduction

Molybdenum is widely used in industrial catalytic processes such as hydrorefining,^1–3^ and currently studied as a promising alternative for highly expensive platinum group metals in processes for producing sustainable energy and reducing environmental pollution.^4–6^ Many recent works describe application of molybdenum sulphides or selenides, nitrides, carbides and phosphides (both supported and unsupported) in the reactions of hydrogen evolution,^7–9^ oxygen reduction,^10,11^ or carbon dioxide reduction.^12^ For both electrochemical and catalytic reactions porous materials with hierarchical systems of interconnected macropores and mesopores are preferable because such structure facilitates diffusion of the reactants. In order to synthesize mesoporous Mo sulfides and nitrides versatile techniques have been proposed such as topotactic transformations of oxides^13,14^ or of hybrid materials.^15^ One of the most popular techniques to control the textural properties is template-based synthesis strategy. To prepare molybdenum sulfide and nitride, templating with silica,^16,17^ polymers and MOFs^18,19^ or biotemplating^20,21^ have been applied. In the majority of templating methods the removal of template must be carried out at the final step, usually by etching. To avoid etching step the use of sacrificial templates is preferable. Carbon nitride has been first used as a sacrificial and reactive template by Antonietti and coll.^22,23^ Upon heating of mesoporous C_3_N_4_ impregnated with metal oxide species chemical reaction occurs that converts oxides to the corresponding nitrides (Al–Ga–N) and (Ti–V–N). Carbon nitride was also applied as a template to obtain N-doped carbon layers with increased porosity by means of thermal treatment at 900 °C.^24,25^ When a process using C_3_N_4_ as a template is carried out in the inert atmosphere, the temperatures as high as 800–900 °C are required to fully transform metal oxide species to nitrides. While suitable to prepare nanoparticles of refractory nitrides, such temperatures are prohibitive for obtaining highly divided materials, because of advanced sintering.

In this work we demonstrate that in the reductive atmosphere carbon nitride can play a role of sacrificial reactive template at much lower temperatures. Moreover, the presence of a transition metal could further decrease the decomposition temperature of C_3_N_4_. This allows obtaining highly divided mesoporous materials, as demonstrated for the case study of MoS_2_ and Mo_2_N materials with hierarchical porosity and advantageous catalytic properties in HDS and HER reactions.

## Experimental

### Materials preparation

To prepare carbon nitride (C_3_N_4_), typically 30 g of urea was placed in a Pyrex tube, covered with a Pyrex cap and heated in static air for 2 h at 550 °C, with heating rate 10 °C min^−1^. Ammonium heptamolybdate (AHM) was supported onto carbon nitride by incipient wetness impregnation from aqueous solution (10% wt. AHM/C_3_N_4_). To carry out the treatments in reductive atmosphere, weighted amount of 10% wt. AHM/C_3_N_4_ (*ca.* 0.8 g) was placed in a Pyrex reactor and treated in a reactive gas mixture (NH_3_/H_2_ or H_2_S/H_2_) for 2 h at 550 °C with heating rate 5 °C min^−1^. The obtained samples are designated as Mo_2_N–CN and MoS_2_–CN, respectively. The solid obtained after heating of AHM/C_3_N_4_ in pure nitrogen flow was also characterized as a reference.

### Characterizations

Textural properties of the C_3_N_4_ template and Mo-containing samples were studied by N_2_ adsorption–desorption volumetry at −196 °C on a Micrometrics ASAP 2010 device. Pore distributions have been calculated using BJH equation. The samples were outgassed before the measurements at 400 °C for 2 h. Phase composition was studied by X-ray diffraction (XRD) on a Bruker D8 Advance A25 diffractometer with CuKα emission. The phases were identified by comparison with JCPDS standards database. Phase composition was quantified using Rietveld refinement as implemented in the Philips XPert software. CHONS analysis was performed on a Thermo Fisher Flash 2000 device. Transmission Electron Microscopy (TEM) images were obtained on a JEOL 2010 instrument at 200 KV. TEM images were analyzed using Digital Micrograph Gatan program package. Temperature-programmed reduction (TPR) was carried out in a quartz reactor. The samples (*ca.* 0.01 g) were linearly heated under a hydrogen flow (50 ml min^−1^) from room temperature to 1050 °C (heating rate 5 °C min^−1^). The gases evolved upon reduction were detected by means of Thermo Fischer quadrupole mass-spectrometer. Thermogravimetric analysis (TGA) was carried out on a SETARAM device. A weighted amount of sample powder (5–10 mg) was placed in an alumina crucible and heated in nitrogen flow from room temperature to 800 °C at a 10 °C min^−1^ rate (NB: cyan and HCN released upon TPR and TGA experiments are toxic and should be neutralized at the reactor outlet).

### Catalytic tests

The catalysts were tested in thiophene HDS right after their preparation: 40 mg of the sample was placed in a quartz reactor in continuous flow of H_2_ (50 ml min^−1^) passed through a bubbler with thiophene. HDS was studied at 320, 330, 340 °C, respectively. The products were analyzed by gas chromatography on an Agilent 7820A device.

Electrochemical hydrogen evolution reaction (HER) was performed in a three electrode electrochemical cell in Ar-saturated 0.5 M H_2_SO_4_ electrolyte at room temperature. Glassy carbon rotating electrode was used as a working electrode, saturated calomel electrode as a reference electrode and graphite rod as a counter electrode. 10 mg of catalyst was suspended in 400 μl of Nafion (0.5%) and 800 μl of EtOH and treated by ultrasound. 10 μl of the obtained catalytic ink was spread on the working electrode and dried. Activity of the catalysts in HER was measured in the potential range from −800 mV to 100 mV. LSV curves were obtained at 5 mW s^−1^ rate. Tafel slopes were calculated *via* Tafel equation. Electrochemically active surface area (ECSA) was calculated from the non-faradaic parts of CV curves at sweep rates from 50 to 300 mV s^−1^.

## Results and discussion

### Reactivity of C_3_N_4_*vs.* the nature of gas atmosphere and the presence of molybdenum

**Fig. 1:**
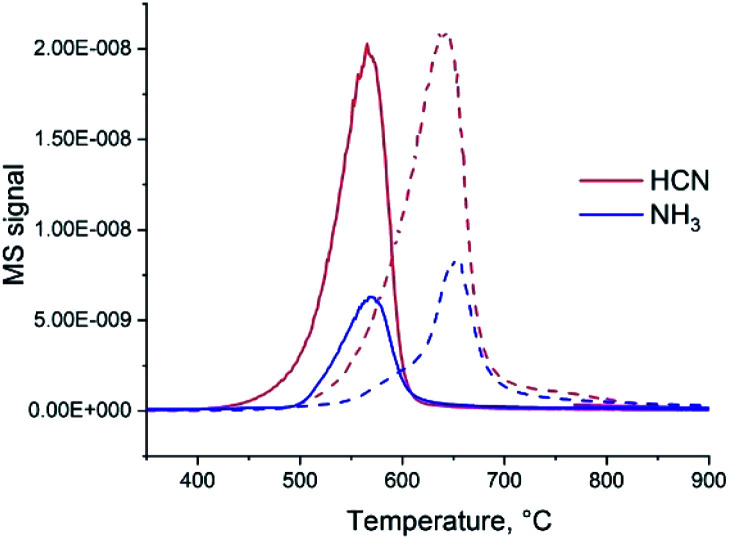
MS signals of gases, released during heating in hydrogen of the C_3_N_4_ and AHM/C_3_N_4_ samples. The solid lines correspond to AHM/C_3_N_4_ and dashed lines to bare C_3_N_4_ (the intensity of MS signals was normalized).

If heated under nitrogen flow, bare carbon nitride starts to decompose approximately at 600 °C and the decomposition completes at 780 °C (Fig. S1†). The mass loss is nearly 100% (the solid completely disappears from the crucible). These results are in good agreement with previous reports of the TGA of C_3_N_4_ carried out in nitrogen.^26^ As shown by mass spectrometry,^27^ gaseous CN and C_2_N_2_ species and N_2_ are formed during the decomposition of C_3_N_4_. Addition of ammonium heptamolybdate (AHM) by impregnation results in the decrease of C_3_N_4_ decomposition temperature by nearly 100 °C (Fig. S1†). This suggests a chemical reaction between AHM and C_3_N_4_ and probably a catalytic effect of formed Mo species on the decomposition. The mass loss is 92%; a solid residual was observed in the crucibles. Changing the gas flow from N_2_ to H_2_ leads to a further decrease of the onset temperature of decomposition (Fig. 1). For bare C_3_N_4_ it shifts to 500 °C and for the AHM/C_3_N_4_ sample it becomes approximately 450 °C. At the same time the nature of the released gases is modified: formation of NH_3_ and HCN occurs in hydrogen flow instead of cyan release previously observed in the inert atmosphere.

Therefore, both addition of Mo species and applying hydrogen flow lead to a decrease of the C_3_N_4_ elimination temperature. Moreover, simultaneous application of H_2_ and addition of AHM provides a synergistic effect. Similar reaction onset temperature of 450 °C is observed for AHM/C_3_N_4_ sample in pure H_2_ (Fig. 1) and in the NH_3_/H_2_ mixture (Fig. S2†).

### Properties of the solid products

**Fig. 2:**
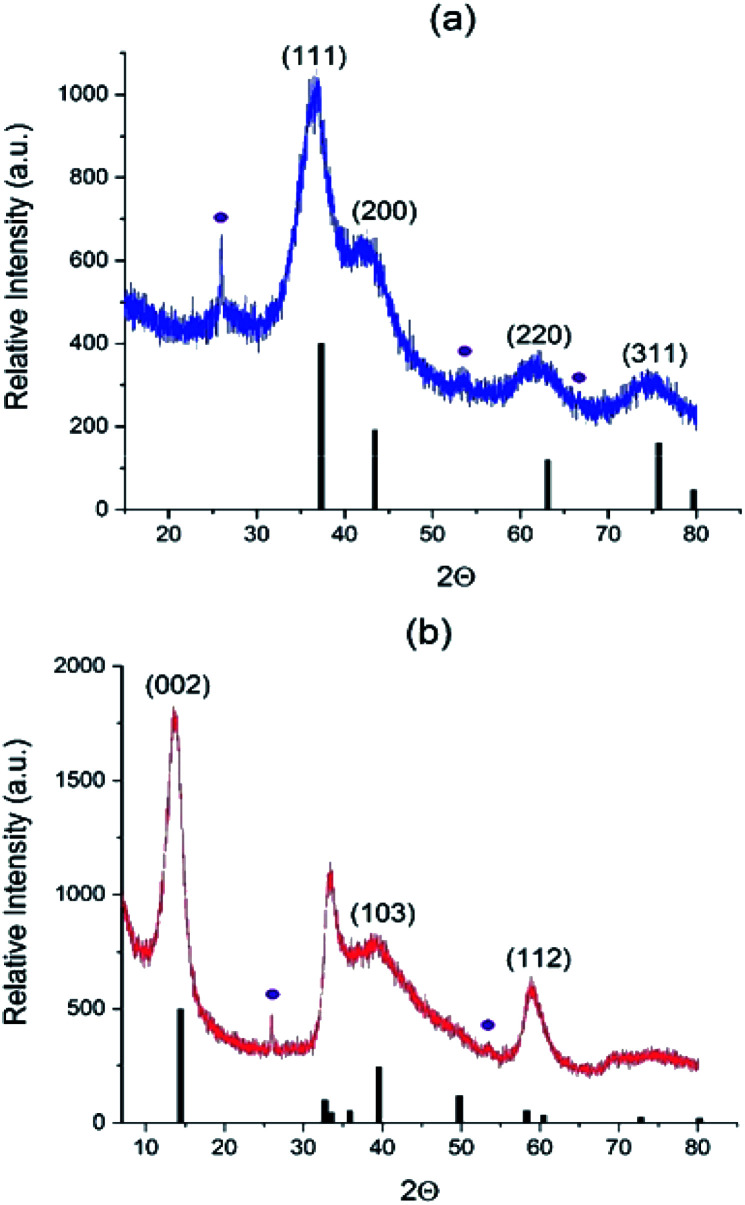
Diffractograms of (a) Mo_2_N–CN (b) MoS_2_–CN. Black bars correspond to (a) Mo_2_N phase (00-025-1366) and (b) 2H–MoS_2_ phase (03-065-0160); violet circles correspond to MoO_2_ impurity (01-078-1070).

We further collected the solid products of AHM/C_3_N_4_ reactions in different atmospheres (N_2_, NH_3_/H_2_ and H_2_S/H_2_) and analyzed their properties. The XRD patterns are shown in Fig. 2 and S3.† In the N_2_ atmosphere highly divided γ-Mo_2_N is formed at 650 °C (Fig. S3†), but it is polluted with MoO_2_ oxide (13% vol. according to Rietveld analysis). However, observing Mo_2_N as a major phase proves that C_3_N_4_ plays the role of nitrogen source and therefore acts as a reactive template. Using NH_3_/H_2_ and H_2_S/H_2_ flows allows obtaining at 550 °C almost pure phases of cubic γ-Mo_2_N and hexagonal 2H–MoS_2_ (Fig. 2), respectively (and small impurity of MoO_2_, 2–4% vol).

In agreement with XRD, chemical analysis shows the S and N content values close to the theory values for the Mo sulfide and nitride, respectively (Table 1). Carbon is almost completely removed from the MoS_2_–CN sample, but is still present in the Mo_2_N–CN (probably as amorphous matter not detected by XRD). Small amount of oxygen in both samples is probably due to partial surface oxidation and due to a contribution from minor MoO_2_ impurity.

**Table 1:** Results of CHONS analysis

Element, wt% sample	N	C	H	S	O
C_3_N_4_	62.1	35.3	1.55	0	0.91
Mo_2_N–CN	6.6	8.4	0.72	0	2.09
MoS_2_–CN	1.7	0.43	0.25	29.0	3.9

Therefore, the use of the reactive gas mixtures (NH_3_/H_2_ or H_2_S/H_2_) allowed us to obtain at 550 °C the materials containing molybdenum nitride or sulfide as major components. We further studied the morphology and the catalytic properties of these materials.

**Fig. 3:**
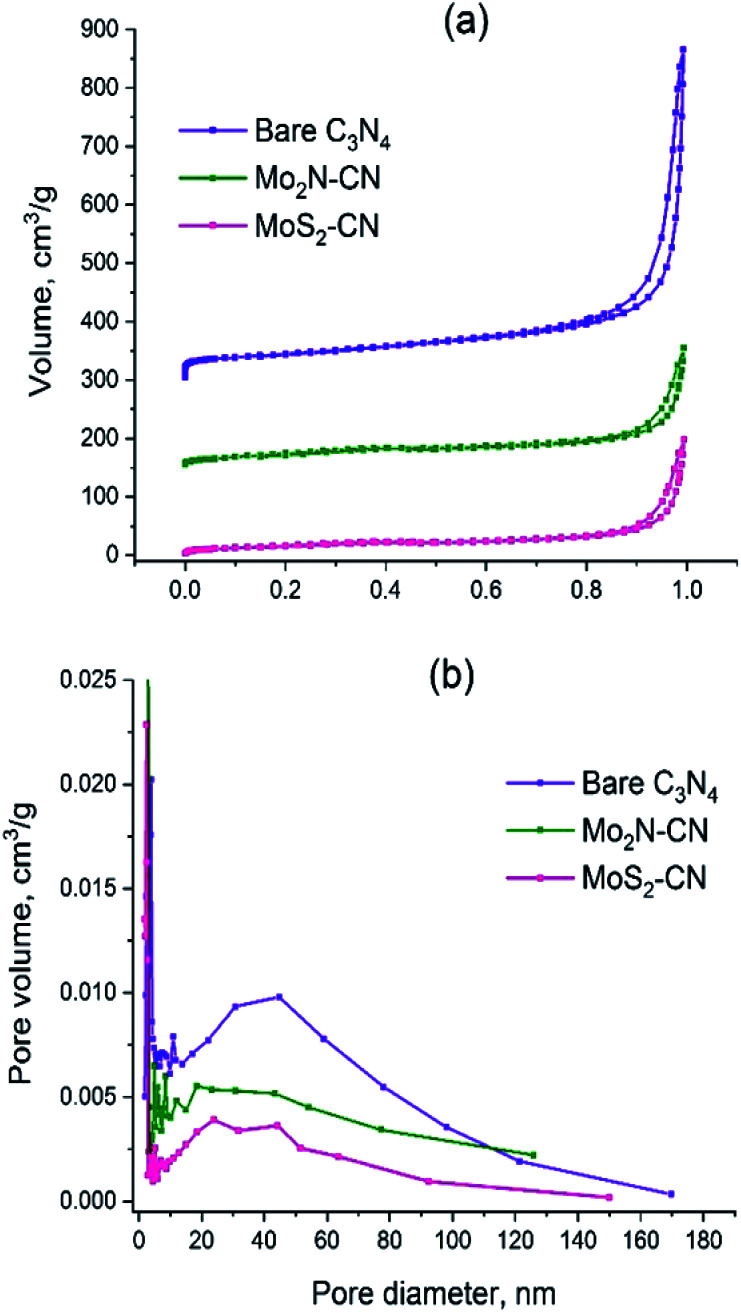
(a) N_2_ adsorption–desorption isotherms for C_3_N_4_, Mo_2_N–CN and MoS_2_–CN; (b) BJH pore distributions for C_3_N_4_, Mo_2_N–CN, MoS_2_–CN.

The C_3_N_4_ template has the specific surface area (*S*_BET_) 157 m^2^ g^−1^ and pore volume 0.87 cm^3^ g^−1^ (Table 2). The isotherm shape corresponds to type II (IUPAC classification) which is characteristic of the macroporous materials (Fig. 3a). Pore size distribution is broad and has a polymodal shape (Fig. 3b), the pores with diameters between 30 and 50 nm being the most abundant ones. The amount of micropores is rather low; the narrow peak at pore radius *ca.* 2 nm is an artifact caused by cavitation-induced evaporation (Fig. 3b).

**Table 2:** Textural properties of the samples

Sample	*S*_BET_, m^2^ g^−1^	Pore volume, BJH, cm^3^ g^−1^
Bare C_3_N_4_	157	0.87
Mo_2_N–CN	96	0.32
MoS_2_–CN	64	0.31

The isotherms of Mo_2_N–CN and MoS_2_–CN have similar shape to that of the parent C_3_N_4_ material, but the pore volumes and the specific surface areas are smaller, obviously because of higher density of the corresponding molybdenum compounds (Table 2). Comparison of isotherms and BJH pore size distributions suggests that Mo_2_N–CN and MoS_2_–CN replicate the main features of the pore structure of C_3_N_4_ template and possess hierarchical mesoporosity. This is an important finding since mesoporous materials of this type are highly demanded and not readily available. Indeed, both Mo_2_N and MoS_2_ obtained by conventional solid–gas reaction techniques are microporous. Bulk Mo_2_N prepared *via* widely used Volpe and Boudart TPR method possesses high specific surface area (around 200 m^2^ g^−1^), but pore size smaller than 30 Å, fully lying in the microporosity range.^28,29^ Similarly, MoS_2_ synthesized by conventional techniques such as ammonium thiomolybdate (ATM) decomposition possesses low pore volume and considerable microporosity.^30^

**Fig. 4:**
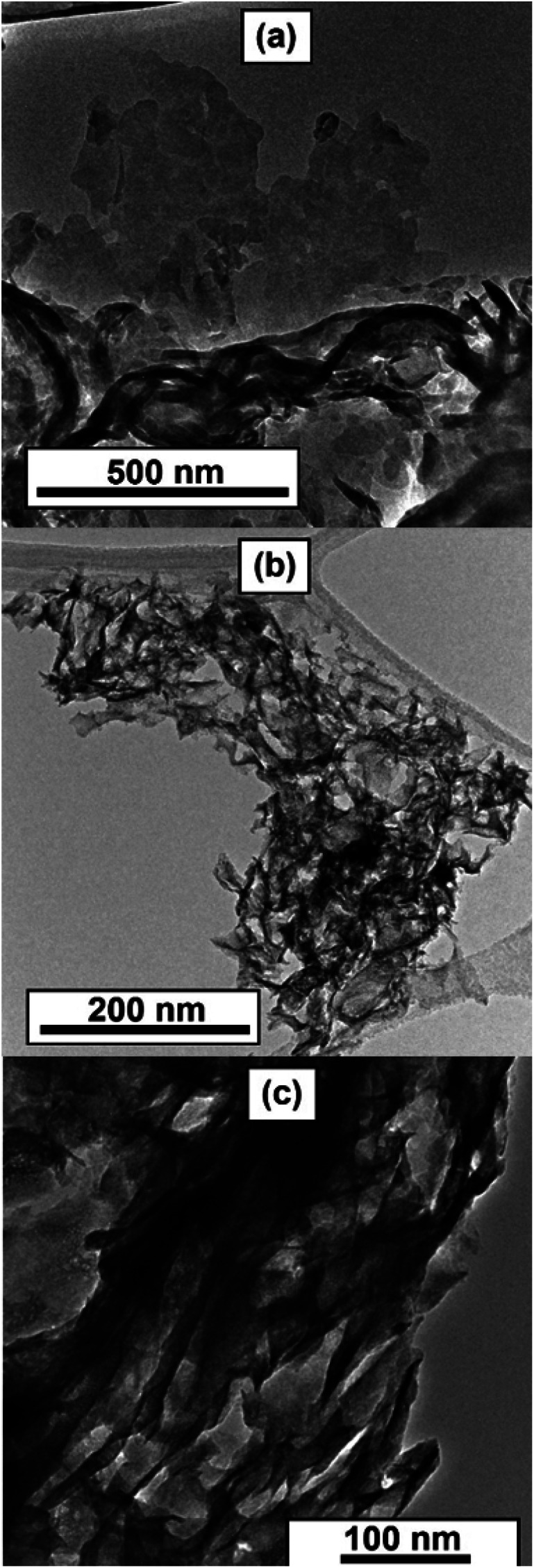
Low magnification TEM images of (a) C_3_N_4_, (b) Mo_2_N–CN, (c) MoS_2_–CN.

Transmission electron microscopy (TEM) study corroborates the results of other characterizations and provides additional insights into the morphology of the solids. Low-magnification TEM reveals lamellar morphology of the C_3_N_4_ template (Fig. 4a and S5†). The layers of carbon nitride are randomly bent, having a rag-like aspect. The morphology of the reaction products Mo_2_N–CN and MoS_2_–CN bears a significant similarity to the parent template. Lamellar morphology and open porosity with convex macropores were observed (Fig. 4b and c; see also video in the ESI†).

**Fig. 5:**
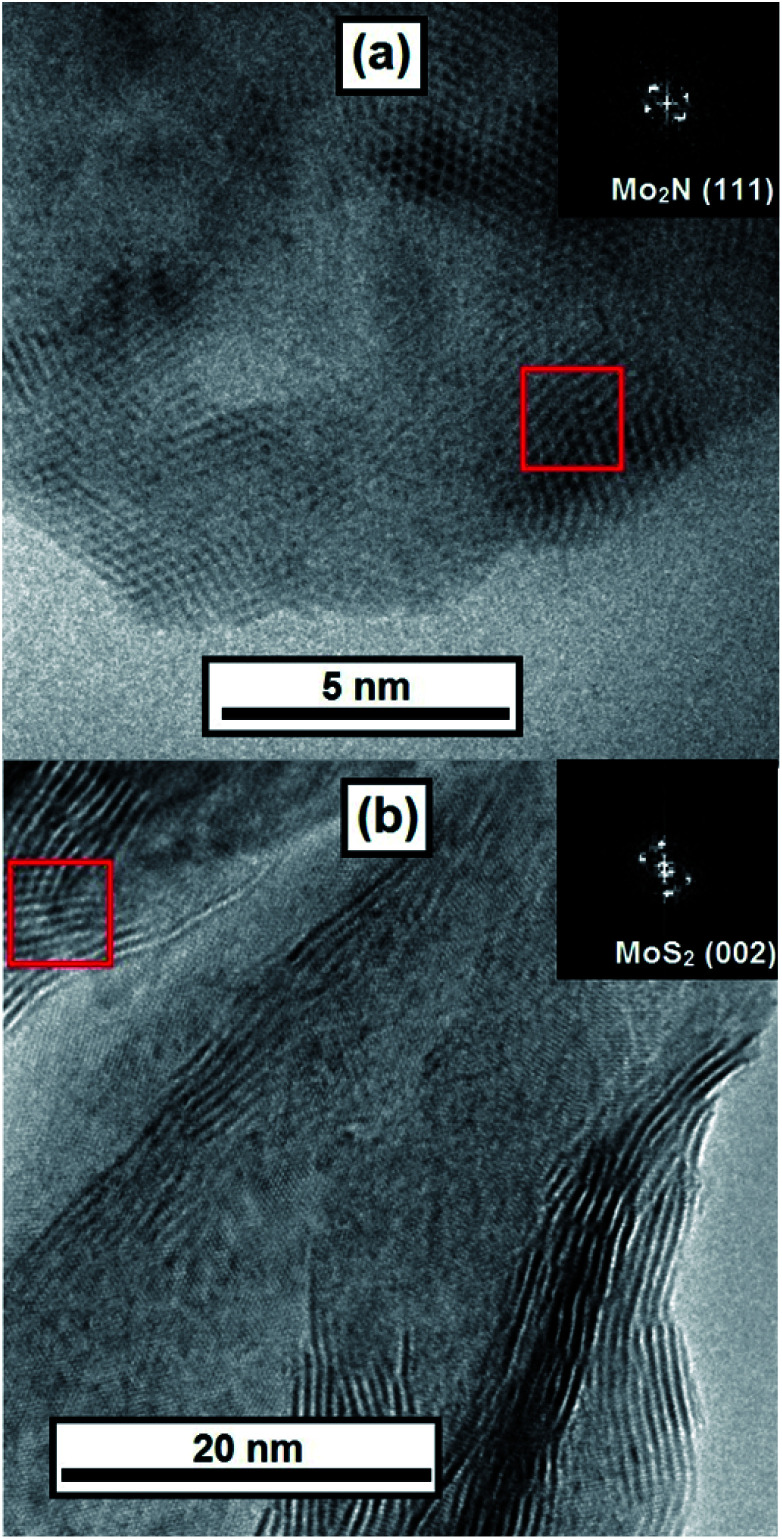
TEM images of (a) Mo_2_N–CN and (b) MoS_2_–CN; insets – digital diffraction for zones marked by red squares.

At higher magnifications stacked layers of MoS_2_ become visible in the MoS_2_–CN sample (Fig. 5b) and several nm-size Mo_2_N particles were observed in the Mo_2_N–CN solid (Fig. 5a).

Interplanar distances 0.205 nm and 0.242 nm correspond respectively to the (2 0 0) and (1 1 1) planes of γ-Mo_2_N. In the MoS_2_–CN sample the measured interplanar distance is 0.67 nm, corresponding to the theory value for (0 0 2) plane of MoS_2_ (0.62 nm) and to the measured XRD peak position (0.65 nm). Note that because of the slabs bending and stacking defects, the measured interplane distance in the nanoscopic MoS_2_ samples is often greater than in the bulk molybdenite.^31^

Overall, the obtained mesoporous Mo_2_N and MoS_2_ materials replicate the hierarchical porous structure and lamellar morphology of the template. Mo_2_N–CN and MoS_2_–CN were further tested in the model reactions of gas-phase thiophene HDS and liquid-phase HER.

### Catalytic properties

**Fig. 6:**
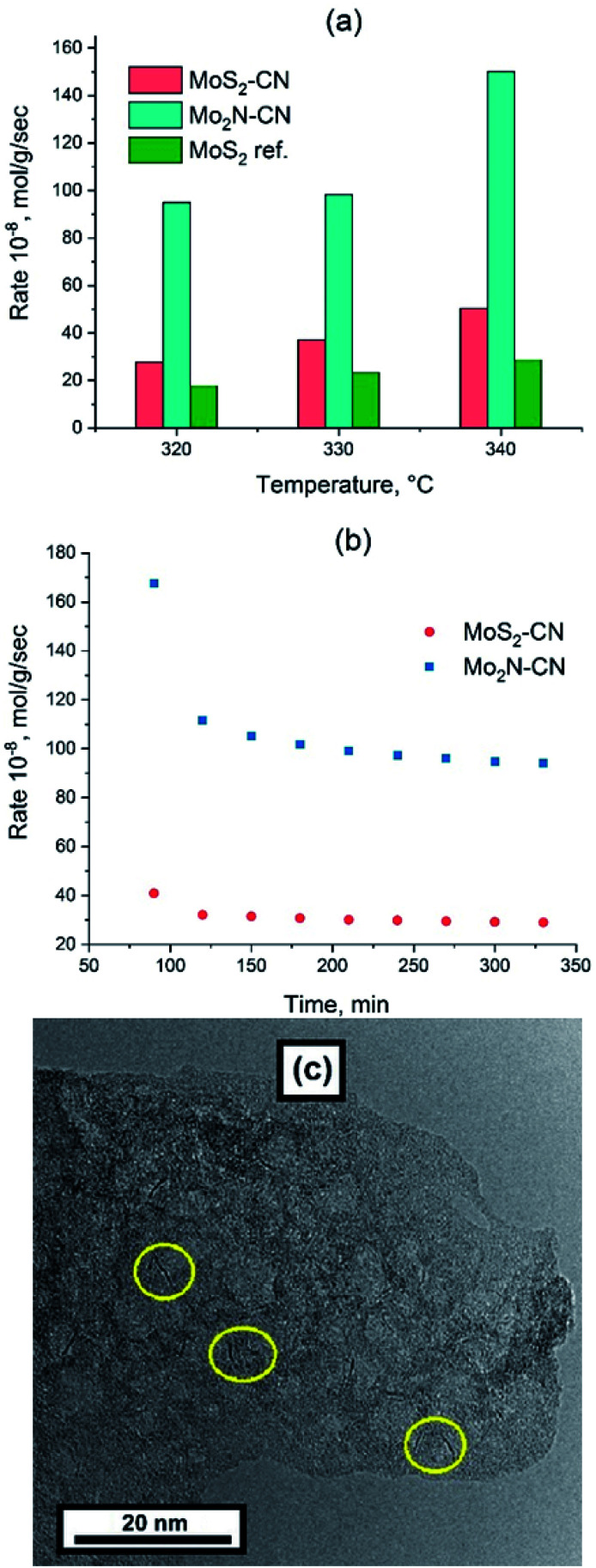
(a) Steady state thiophene conversion rate *vs.* temperature for Mo_2_N–CN, MoS_2_–CN and MoS_2_ bulk reference; (b) time dependence of thiophene conversion rate at 320 °C for Mo_2_N–CN and MoS_2_–CN; (c) TEM image of Mo_2_N–CN after HDS, yellow circles indicate the MoS_2_ slabs.

Thiophene HDS rates are shown in Fig. 6 in comparison with the benchmark ATM–MoS_2_ reference. The Mo_2_N–CN sample demonstrates one of the highest HDS activities reported for the non-promoted Mo catalysts, being at least five times more active than the ATM–MoS_2_ bulk reference and also much more active than bulk MoS_2_ solids from our previous works that were tested in the HDS reaction under the same conditions.^32^ (Fig. 6a).

The evolution of HDS activity *versus* time was significantly different for sulfide and nitride samples. At 320 °C the nitride catalyst loses 50% of its activity during the first hour on-stream, whereas MoS_2_–CN sulfide was deactivated much less (Fig. 6b). The Mo_2_N–CN solid after the HDS test preserved opened porous morphology (Fig. 6c). However, at high resolution short (1–3 nm) MoS_2_ single slabs were observed. Therefore, partial sulfidation of molybdenum nitride occurred at the surface of pores. Steady state HDS activity can be attributed to these slabs, very short due to confinement in the pores of Mo_2_N matrix. In agreement with our findings, it was reported earlier that the initial thiophene HDS activity of γ-Mo_2_N/Al_2_O_3_ is high, but during the reaction rapid sulfidation of the surface occurs and the steady state activity is defined by the MoS_2_ slabs formed on the surface.^33^

Formation of MoS_2_ occurs *via* sulfidation of the passivating surface oxide layer rather than a direct nitride-to-sulfide transformation.^34^ Exceptionally high apparent HDS rate during the initial period might be due to high intrinsic activity of Mo_2_N, but might be also related to the sulfur uptake by nitride.

The MoS_2_–CN sample demonstrates lower HDS activity than the Mo_2_N–CN one, but still higher than the ATM–MoS_2_ reference (Fig. 6a). Indeed, on the TEM images of MoS_2_–CN, extended (>5 nm) and stacked layers of MoS_2_ are mostly present (Fig. 5b). Lower dispersion of MoS_2_ slabs and therefore lower amount of the active edge structures explains its lesser activity in comparison with Mo_2_N–CN. The selectivity distributions of HDS products are similar to those observed earlier for the non-promoted Mo sulfide catalysts (Fig. S6†).^30,32^ Beside C_4_ HDS products, small amounts of methane were detected. Production of methane might be due to partial surface oxidation of Mo_2_N and MoS_2_ with formation of Mo oxide species that possess acidic sites, able to perform cracking.

**Fig. 7:**
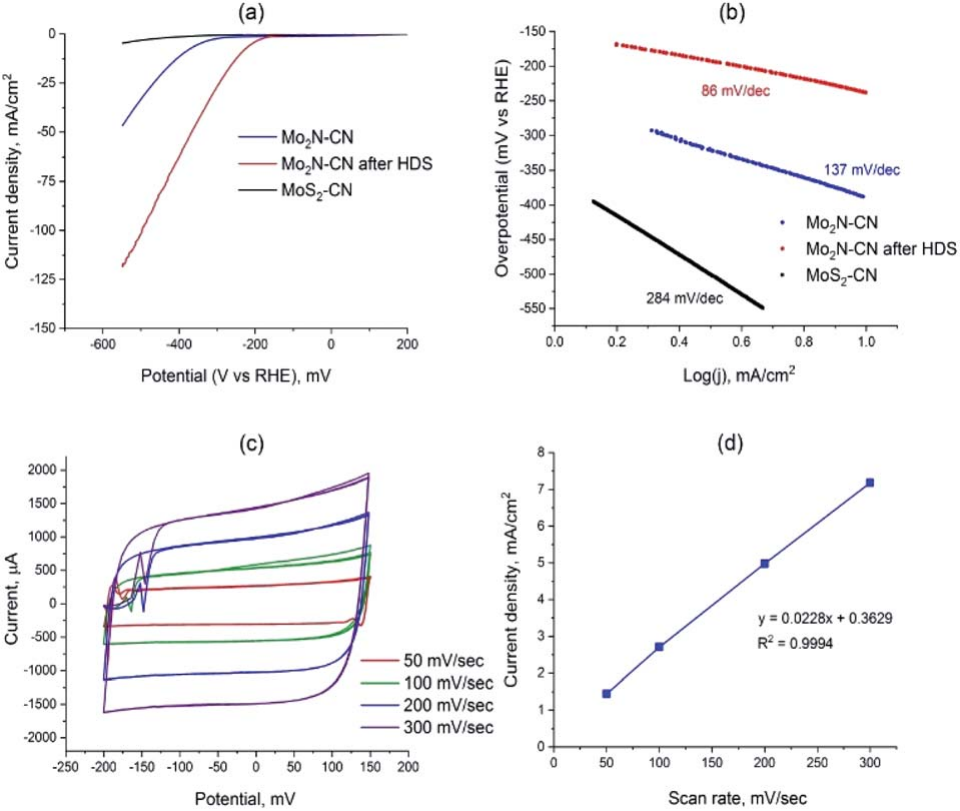
(a) LSV curves of Mo_2_N–CN, Mo_2_N–CN after HDS and of MoS_2_–CN; (b) Tafel slopes for Mo_2_N–CN, Mo_2_N-CN after HDS and MoS_2_–CN; (c) cyclic voltammograms (CV) for Mo_2_N–CN after HDS, measured at the scan rates 50, 100, 200, 300 mV s^−1^; (d) linear dependence between the current density and the scan rates.

To access the potential of electrochemical applications, Mo_2_N–CN (fresh and after HDS test) and MoS_2_–CN were tested in the electrochemical HER in 0.5 M H_2_SO_4_. The MoS_2_–CN material demonstrates poor HER activity, in accordance with its moderate HDS catalytic performance (Fig. 7a). Low activity of bulk 2H–MoS_2_ was previously reported in the literature.^35^ The Mo_2_N based samples demonstrate good activity, the sample taken after HDS being more active than the initial nitride. The overpotential for Mo_2_N–CN after HDS at 10 mA cm^−2^ is much lower (239 mV) than that of the initial nitride sample (390 mV) (Fig. 7a). The calculated Tafel slopes for three tested samples are 86 mV dec^−1^ for Mo_2_N–CN after HDS, 137 mV dec^−1^ for Mo_2_N–CN and only 284 mV dec^−1^ for MoS_2_–CN (Fig. 7b). The obtained values of Tafel slopes for Mo_2_N based catalysts indicate that HER reaction follows Volmer–Heyrovsky mechanism. For the sample Mo_2_N–CN collected after HDS test, that shows the highest HER performance, the electrochemical surface area (ECSA) calculated from the non-faradaic CV at different scan rates is 136 m^2^ g^−1^ (Fig. 7c and d). This value is close to the BET surface area and attests good availability of the surface for the electrochemical reaction.

The superior HER catalytic performance of the Mo_2_N–CN sample after HDS text might be associated with high activity of short MoS_2_ slabs on the surface of Mo_2_N, formed during the HDS reaction due to sulfidation of the catalyst surface by the released H_2_S. Oppositely to the metallic Mo_2_N, 2H–MoS_2_ is a semiconductor, so it possesses relatively low electric conductivity^36^ and, therefore, lower activity in HER, whereas the active sites for these two materials are similar (MoS_2_ slabs edges). It appears that mesoporous Mo_2_N decorated with MoS_2_ fringes provides optimal structure for an efficient HER catalyst. Thus in ref. 37 it was suggested that if MoS_2_ edges are in a tight contact with the surface of Mo_2_N, then the electronic structure of Mo sites located at the interface could be tuned, boosting HER catalytic performance. Recently, in the same lines, for chemically similar W sulfide it was shown that metallic W particles surrounded with WS_2_ show high HER activity.^38^ The interface between MoS_2_ n-type semiconductor, and Mo_2_N would create ohmic or Schottky contact, depending on the difference between the work functions of two materials. For the similar Mo–MoS_2_ contact, low Schottky barrier was observed that potentially provides easy charge injection from current collector to the catalyst sites.^39^ For the Mo_2_N–MoS_2_ interface, both experiment and DFT analysis show that an electric field is created at the interface between Mo_2_N and MoS_2_ that facilitates charge transfer.^40^

## Conclusions

Mesoporous MoS_2_ and Mo_2_N materials replicating the hierarchical mesoporous morphology of C_3_N_4_ were prepared using a simple topotactic solid–gas reaction. Due to the advantageous morphology these solids are well adopted for the applications in the heterogeneous catalysis and in the electrocatalysis. The most active catalyst in both HDS and HER reactions is mesoporous molybdenum nitride decorated at the surface by small MoS_2_ fringes. The HDS performance of this sample is one of the highest ever reported for the non-promoted Mo systems.

Due to application of the reactive gases, our synthetic approach extends the potential use of C_3_N_4_ as a template towards considerably lower temperatures and to the materials other than nitrides. The advantage of this method is its utter simplicity. Indeed, carbon nitride support is impregnated with ammonium heptamolybdate according to the standard procedure, commonly applied for the preparation of heterogeneous catalysts. Further activation with H_2_S/H_2_ or NH_3_/H_2_ is also carried out by following the standard procedures that are used to prepare sulfide and nitride catalysts. Unlike in many other template-based techniques there is no template leaching steps. Obviously, mesoporous sulfides and nitrides of other metals (such as V, W, Cr) and their combinations could be prepared using this approach under relatively soft conditions. By the same token, preparation of mesoporous phosphides or borides could be considered, by means of the reductive treatment of phosphates or borates supported on C_3_N_4_. Beside catalysis, other applications could be considered in the fields where materials with open porosity are demanded, such as pseudo capacitors, electrodes or sensors.

## Author contributions

DR: writing, materials preparation, catalytic tests, PA: conceptualising, materials characterizations, writing.

## Conflicts of interest

There are no conflicts to declare.

## Supplementary Material

RA-011-D1RA03657B-s001
