# Supplementary material for: Carbon nitride used as a reactive template to prepare mesoporous molybdenum sulfide and nitride
Source: RSC Adv. 2021 Jun 18;11(35):21678–84. doi: 10.1039/d1ra03657b (PMC9034131; doi:10.1039/d1ra03657b)
Supplement: RA-011-D1RA03657B-s001 [file RA-011-D1RA03657B-s001.pdf]

Electronic Supplementary Material (ESI) for RSC Advances.
This journal is © The Royal Society of Chemistry 2021

SUPPLEMENTARY INFORMATION

Carbon nitride used as a reactive template to prepare mesoporous molybdenum sulfide and nitride.

Daria Ryaboshapka, Pavel Afanasiev

Univ Lyon, Univ. Claude Bernard Lyon 1, CNRS, UMR5256, IRCELYON, F-69626, Villeurbanne, France.

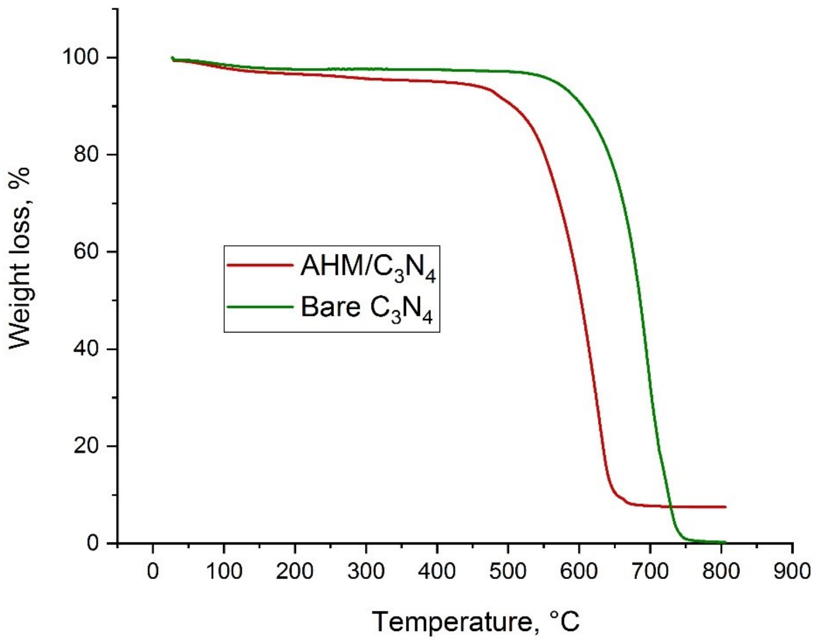

Fig. S1 TG curves for AHM/C_3N_4 and bare C_3N_4

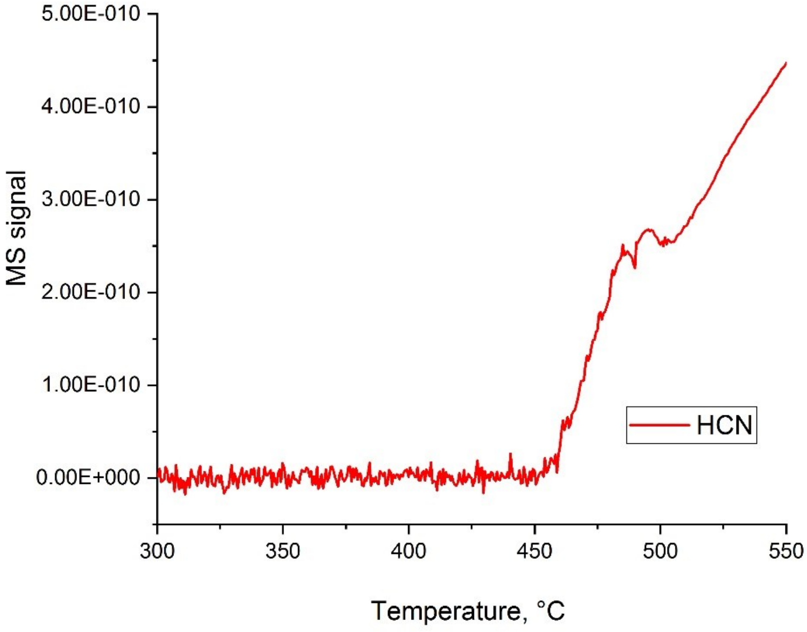

Fig. S2 Mass spectrum recorded during preparation of Mo_2N-CN

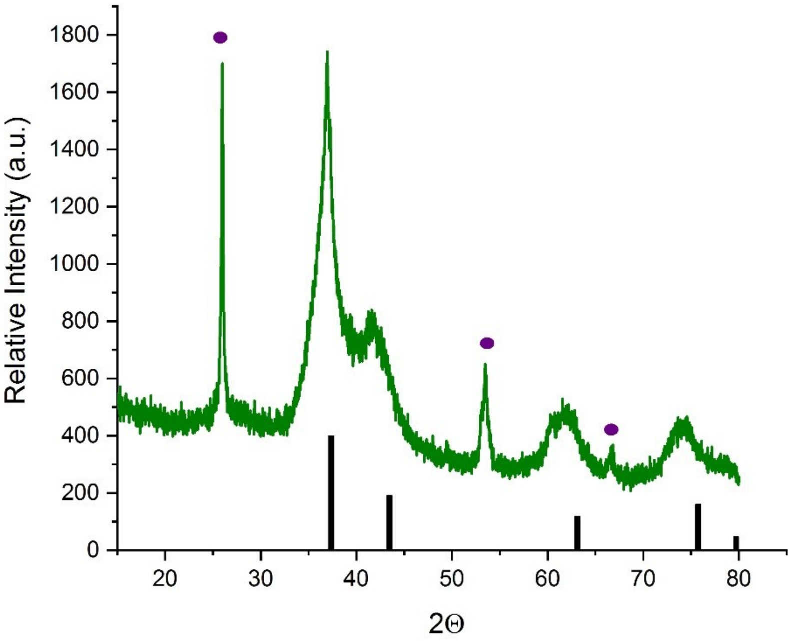

Fig. S3 Diffractogram of the sample prepared from AHM/C_3N_4 in N_2 flow at 650 °C, black bars correspond to Mo_2N phase (00-025-1366) and violet circles correspond to MoO_2 phase (01-078-1070)

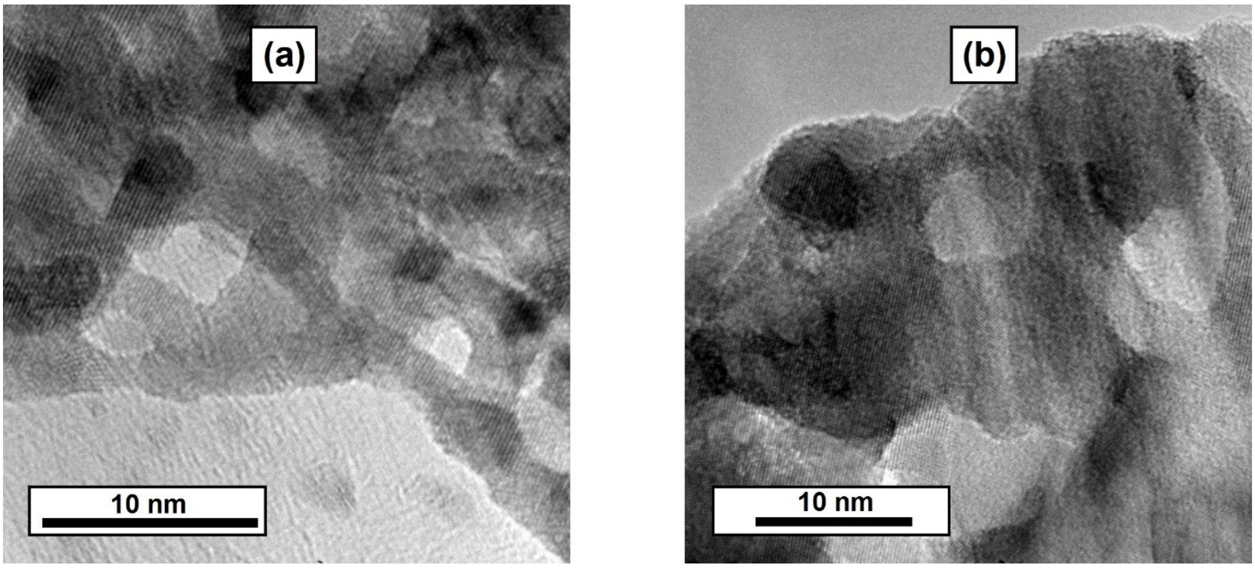

Fig. S4 TEM microphotographs of reference sample (AHM/C_3N_4 treated in N_2 flow at 650 °C)

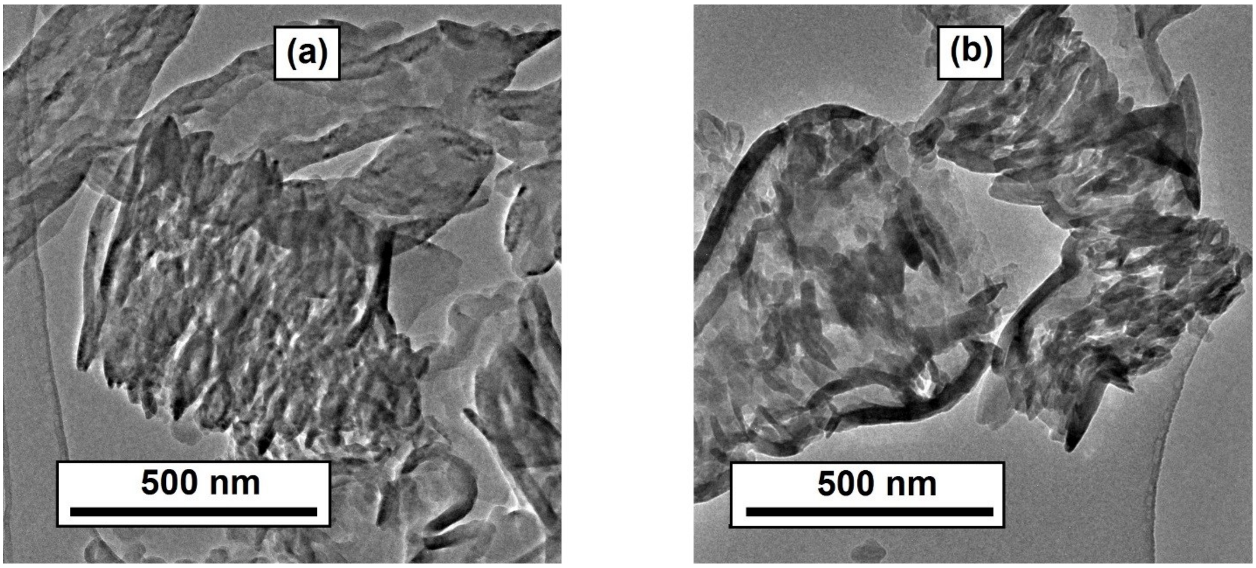

Fig. S5 Low magnification TEM images of C_3N_4

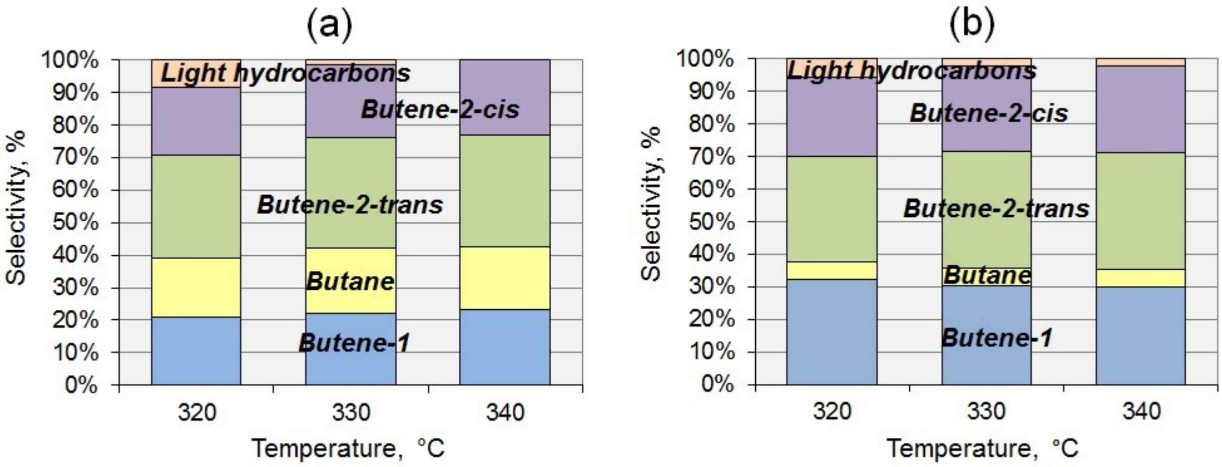

Fig. S6 Selectivity distributions in HDS for (a) Mo_2N-CN, (b) MoS_2-CN

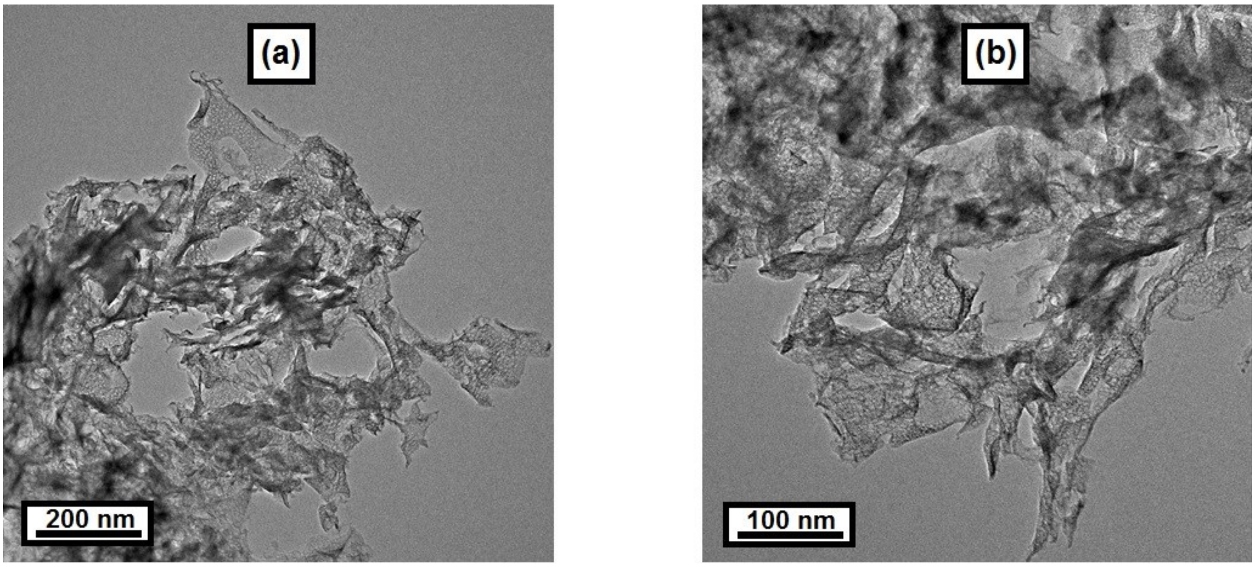

Fig. S7 TEM images of Mo_2N-CN after HDS
